# Therapeutic Challenges in Total Situs Inversus Associated with Sick Sinus Syndrome, Ventricular Arrhythmias, and Heart Failure with Preserved Ejection Fraction: Narrative Review and Case Report

**DOI:** 10.3390/jcdd12110419

**Published:** 2025-10-22

**Authors:** Cristina Tudoran, Mariana Tudoran, Dragos Cozma, Cristina Văcărescu, Ahmed Abu-Awwad, Simona-Alina Abu-Awwad, Dragos Cătălin Jianu, Florica Voita-Mekeres

**Affiliations:** 1 Department VII, Internal Medicine II, Discipline of Cardiology, University of Medicine and Pharmacy “Victor Babes” Timisoara, E. Murgu Square, Nr. 2, 300041 Timisoara, Romania; 2 Center of Molecular Research in Nephrology and Vascular Disease, Faculty of the Victor Babes University of Medicine and Pharmacy, E. Murgu Square, Nr. 2, 300041 Timisoara, Romania; 3 County Emergency Hospital “Pius Brinzeu”, L. Rebreanu, Nr. 156, 300723 Timisoara, Romania; ahm.abuawwad@umft.ro (A.A.-A.); alina.abuawwad@umft.ro (S.-A.A.-A.); 4 Cabinet Medical Dr. Tudoran, Str. Glad, Nr. 35 A, 300480 Timisoara, Romania; 5 Department of Cardiology, “Victor Babeș” University of Medicine and Pharmacy, E. Murgu Square, Nr. 2, 300041 Timisoara, Romania; dragos.cozma@umft.ro (D.C.); vacarescu.cristina@umft.ro (C.V.); 6 Institute of Cardiovascular Diseases Tmimisoara, 300310 Timisoara, Romania; 7 Research Center of the Institute of Cardiovascular Diseases, 300310 Timisoara, Romania; 8 Department XV—Discipline of Orthopedics—Traumatology, “Victor Babes” University of Medicine and Pharmacy, E. Murgu Square, Nr. 2, 300041 Timisoara, Romania; 9 Research Center University Professor Doctor Teodor Șora, “Victor Babes” University of Medicine and Pharmacy, E. Murgu Square, Nr. 2, 300041 Timisoara, Romania; 10 Department XII—Discipline of Obstetrics and Gynecology, “Victor Babes” University of Medicine and Pharmacy, E. Murgu Square, Nr. 2, 300041 Timisoara, Romania; 11 Department VIII, Clinic of Neurology I, “Victor Babeș” University of Medicine and Pharmacy, E. Murgu Square, Nr. 2, 300041 Timișoara, Romania; jianu.dragos@umft.ro; 12 Centre for Cognitive Research in Neuropsychiatric Pathology (Neuropsy-Cog), Faculty of Medicine, “Victor Babeș” University of Medicine and Pharmacy, E. Murgu Square, Nr. 2, 300041 Timișoara, Romania; 13 Morphological Disciplines Department, Faculty of Medicine and Pharmacy, University of Oradea, 410087 Oradea, Romania; mekeres_florica@yahoo.com; 14 Psychiatry Department, County Clinical Emergency Hospital of Oradea, 410169 Oradea, Romania

**Keywords:** total situs inversus, ventricular arrhythmias, sick sinus syndrome, pacemaker implantation, heart failure with preserved ejection fraction

## Abstract

Background: Total situs inversus (TSI) is a rare genetic anomaly, and approximately half of the affected individuals also have other associated cardiovascular anomalies. Thus, the concomitance of conduction and rhythm disturbances is seldom described in the medical literature. Methods: We searched the medical literature for similar cases published as full text, in English, on Clarivate, PubMed, and Google Scholar between 2016 and 2025. Results: We found 9 reports on TSI patients also having sick sinus syndrome (SSS) associated with rhythm disturbances, mainly atrial fibrillation, raising diagnostic and procedural challenges due to the anatomical anomalies requiring a peculiar approach. We describe the case of a 43-year-old woman diagnosed with TSI associated with ventricular arrhythmias in 2015 who experienced SSS requiring the implantation of a pacemaker during 10 years of follow-up but continued to have frequent episodes of nonsustained ventricular tachycardia (NSVT), raising multiple diagnoses and therapeutic challenges. After developing heart failure with preserved ejection fraction, she received guideline-adjusted treatment and, surprisingly, her clinical status improved, and NSVT diminished in frequency and then disappeared. Conclusions: Highlighting TSI′s clinical implications, often associated with other cardiovascular abnormalities, is important for an accurate diagnosis and adapted therapeutic management, considering the procedural challenges and potential complications.

## 1. Introduction

Total situs inversus (TSI) is a genetic abnormality characterized by a complete, mirror-like transposition of the intrathoracic and intra-abdominal organs. This condition is quite rare, with approximately 1 in 10,000 or even 15,000 births [1], and is more frequently reported in males (1.5:1). Although a particular genetic defect is not specified, available data indicate autosomal recessive and, sometimes, X-linked inheritance. It is assumed that approximately half of the individuals diagnosed with TSI also have other associated cardiovascular malformations [1].

Mentioned a long time ago in animals by Aristotle, Küchenmeister was the first to diagnose TSI by physical examination in four living people [1]. He documented these findings with drawings in 1888 and named this anatomical situation “situs viscerum transversus” [1]. The first researcher who demonstrated this organ transposition by X-ray was Vehsemeyer in 1897 [2]. From this moment, imagistic methods have become the most important diagnostic tools, although a suspicion of TSI could also arise during a thorough medical exam or in emergent surgical intervention. Even a routine electrocardiogram (ECG) could evidence the inversion of the electrical axis and waves [3]. The principal diagnostic methods for TSI are radiography and ultrasonography; however, thoracic and abdominal computed tomography (CT) or magnetic resonance imaging (MRI) are preferred for an accurate diagnosis and the assessment of all anatomical abnormalities [4]. It is assumed that several other congenital cardiovascular malformations are frequently associated with this condition [3,5,6,7].

During surgical and interventional procedures, therapeutic difficulties may arise, especially when performed in emergency, without a proper previous diagnosis of the associated anatomical abnormalities. Considering that most physicians are right-handed and procedural protocols are often developed for this situation, this can represent an issue. That is probably the reason why most scientific articles published on this topic debate procedural difficulties and peculiar situations encountered in patients with TSI [8,9].

We aimed to research the rare association of TSI with conduction disturbances, such as sick sinus syndrome (SSS), who also have associated ventricular arrhythmias (VA) [10,11]. In this paper, we present a particularly rare case of a 43-year-old woman diagnosed with TSI, VA, and SSS in the absence of structural heart diseases diagnosed by imagistic methods and genetic testing, who raised multiple diagnostic and therapeutic challenges, requiring repeated invasive procedures and a pacemaker implantation. During a 10-year follow-up, she developed heart failure with preserved ejection fraction (HFpEF) requiring specific medical therapy and had a peculiar evolution of HFpEF and VA. This case presentation draws attention to the fact that serious rhythm and conduction disturbances may occur in TSI patients even in the absence of evident structural heart alterations and that new drugs used to treat HFpEF could have unexpected beneficial effects that need to be further studied.

## 2. Case Report

### 2.1. Initial Presentation

**Figure 1:**
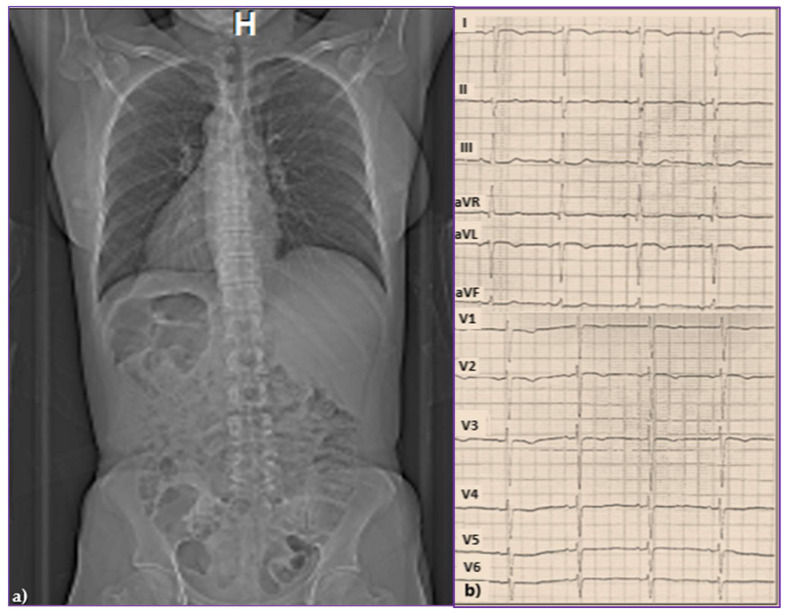
(**a**) Chest and abdominal computed tomography evidencing total situs inversus and (**b**) standard 12-lead surface electrocardiogram.

A 43-year-old woman, already diagnosed with systemic hypertension (SH) and asthma, attended the emergency room in February 2015 for chest pain, palpitations, and high blood pressure (BP) values (170/110 mmHg). In her family history, her father experienced sudden cardiac death at 47 years old. On the chest X-ray, followed by thorax and abdominal CT, TSI was diagnosed through the inverted position “mirror image” of the thoracic and abdominal organs (Figure 1a). On the electrocardiogram (ECG), sinus rhythm 58 beats/minute (b/min), right axis deviation, negative T waves in V1–V3, and premature ventricular beats (PVB) were evidenced, Figure 1b. The 24 h Holter ECG monitoring revealed a mean heart rate (HR) of 47 beats/min with a minimum of 34 beats/min at midday (13:45 h), and frequent isolated PVB classified as Lown class II.

Transthoracic echocardiography (TTE) evidenced normal cardiac cavities, an asynchronous contraction of the interventricular septum, and mild aortic, mitral, and tricuspid regurgitations. Therapy with an angiotensin-converting enzyme (ACE) inhibitor, a calcium channel blocker, a statin, and aspirin was initiated. A small dose of beta-blockers (nebivolol 2.5 mg) was given initially, but after three days, it was suspended because of bradycardia.

### 2.2. Diagnostic of SSS and Pacemaker Implantation

**Figure 2:**
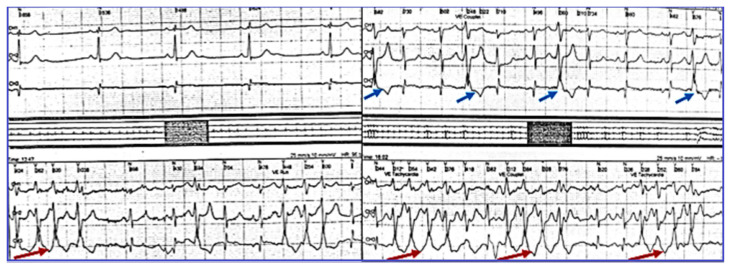
Holter monitoring (derivations DII, DIII, AVF) revealing PVBs, and episodes of unsustained ventricular tachycardia.

The patient remained under observation, and after two years of stable evolution, she experienced again palpitations and dizziness. On the ECG, the persistence of negative T waves in V1–V3 was noticed; there were frequent PVB, often bigeminated, or occurring in doublets or triplets. Holter ECG monitoring revealed 247 episodes of monomorphic, nonsustained ventricular tachycardia (NSVT) of 4–8 beats as illustrated in Figure 2, and sinus bradycardia (average HR = 62, and min = 36 b/min at 14:25 h), confirming the diagnosis of SSS. During the maximal stress test (125 W), the maximum HR was 76 beats/min (44% of her target), revealing a chronotropic incompetence, without VA. Repeated Holter ECG monitoring revealed severe bradycardia (average 29 beats/min, minimum 17 beats/min at 14:54) with 175 sinus pauses longer than 2.4 s, the longest of 6.222 s, rare isolated PVBs, sinus pauses, and idioventricular escape rhythm.

**Figure 3:**
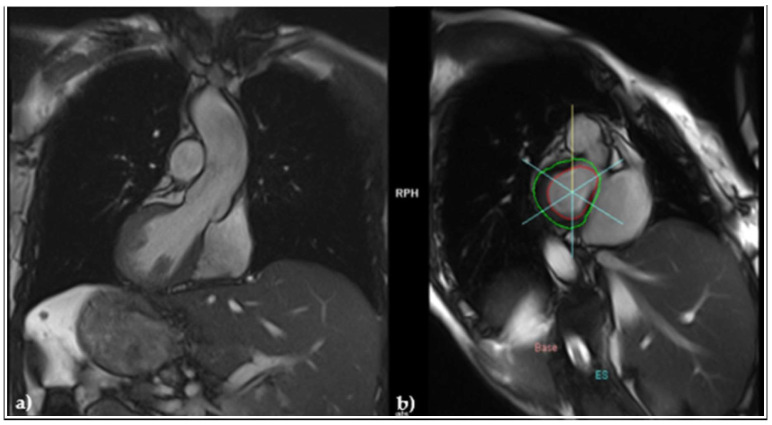
MRI (**a**) frontal view revealing normally sized cardiac cavities; (**b**) sagittal view evidencing and the absence of adipose deposits.

As the patient had a family history of sudden cardiac death (her father died suddenly at 47 years old), an arrhythmogenic right ventricular (RV) cardiomyopathy (ARVC) was suspected. Still, the cardiac magnetic resonance imaging (MRI) evidenced normal cavities with a left ventricular (LV) ejection fraction (EF) of 60% (Figure 3a), and a slightly impaired RVEF, but without adipose deposits (Figure 3b).

In these circumstances, starting from the premise that severe bradycardia may favor the occurrence of NSVT, the implantation of a pacemaker was recommended. A dual-chamber rate-responsive (DDDR) Biotronik pacemaker was inserted in February 2017 by employing a left subclavicular vein access. Subsequently, two Biotronik leads were introduced through the superior vena cava, one in the RV apex, and another in the right atrial appendage. The sensing and threshold parameters were set as appropriate. The device was programmed as DDDR, HR of 60 beats/min, with a long atrio-ventricular delay to allow the intrinsic conduction and minimize RV pacing.

### 2.3. Evaluation of VA, Antiarrhythmic Therapy with Amiodarone and Secondary Effects

Therapy with a beta-blocker (metoprolol succinate 50 mg/day) was associated, and the patient′s clinical evolution was initially favorable. After a while, due to the relapse of NSVT on a new Holter ECG monitoring, amiodarone was resumed at a dose of 200 mg/day. After several months, NSVT reappeared despite antiarrhythmic treatment, requiring the doubling of amiodarone′s dose. Considering that the pacemaker′s testing indicated good stimulation parameters (Avp/s = 220/180 ms) but multiple episodes of NSVT and sustained periods of VT were evidenced on the ECG Holter monitoring, a detailed electrophysiological study (EPS) was performed in November 2017. Programmed ventricular stimulation from standard sites (right ventricular apex and outflow tract), combined with burst pacing and adrenergic provocation using dobutamine, failed to induce sustained ventricular tachycardia (VT). No abnormal electrograms or evidence of arrhythmogenic substrate were identified. Despite these comprehensive maneuvers, no sustained or USVT was induced. High-dose isoproterenol or substrate mapping was not employed, and no late potentials or abnormal electrograms suggestive of an arrhythmogenic substrate were observed. At this moment, no 3D mapping system was used, considering the high cost and limited added value of electroanatomical mapping in the absence of spontaneous or mappable ectopy. The interventional cardiologist encountered the same technical difficulties as during the pacemaker implantation. These findings, along with a structurally normal heart on cardiac MRI, and the patient being oligosymptomatic during these arrhythmias, contributed to the clinical decision against the implantation of an intraventricular cardioverter-defibrillator (ICD) that would have required the implantation of new electrodes and probably of a second device. The patient remained under antiarrhythmic therapy (amiodarone). However, we acknowledge that the absence of VT inducibility does not rule out future arrhythmic risk, particularly in inherited or idiopathic arrhythmia syndromes, and warrants continued follow-up.

In October 2020, she developed autoimmune thyroiditis, and a rigorous endocrinologic follow-up was started, considering the prolonged therapy with amiodarone. Therefore, an episode of hyperthyroidism was diagnosed and was treated accordingly with an antithyroid drug and cessation of amiodarone for six months. Afterwards, because of the presence of VA on the 24 h Holter ECG monitoring (2205 PVB and 16 episodes of NSTV), this therapy was resumed with a dose of 100 mg/day.

In June 2021, type II diabetes mellitus (T2DM) was diagnosed, and treatment with oral glucose-lowering drugs was started. Concomitantly, the patient′s previously diagnosed dyslipidemia worsened with augmentation of LDL cholesterol and triglycerides, which is why alirocumab 150 mg twice/month and fenofibrate 145 mg/day were added to the previous lipid-lowering therapy.

### 2.4. Diagnosis of Heart Failure, Initiation of Guideline-Guided Therapy, and Evolution

**Figure 4:**
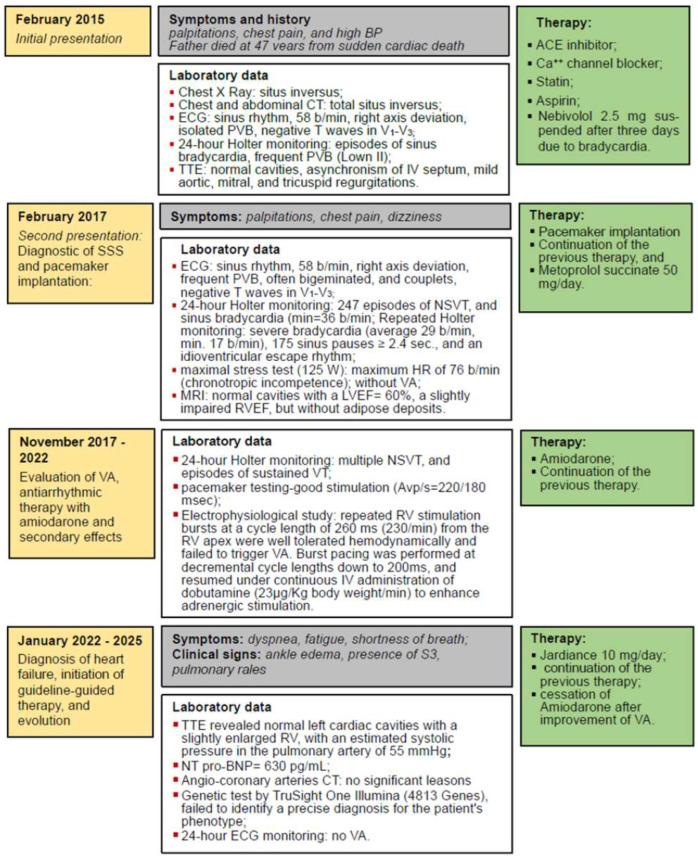
Timeline graphic of the case presentation. Legend: ACE—Angiotensin-converting enzyme; BP—blood pressure; Ca^++^—Calcium; CT—computed tomography; HR—heart rate; IV—interventricular; LVEF—left ventricular ejection fraction; NSVT—non-sustained ventricular arrhythmias; NT pro-BNP—N-terminal pro-brain natriuretic peptide; RV—right ventricle; RVEF—right ventricular ejection fraction; PVB—premature ventricular beats; SSS—sick sinus syndrome; TTE—transthoracic echocardiography; VA—ventricular arrhythmias; VT—ventricular arrhythmia.

In January 2022, she developed symptoms (dyspnea, fatigue, shortness of breath) and clinical signs (ankle edema, presence of the third heart sound, basal pulmonary rales) suggestive of heart failure (HF). TTE revealed normal cardiac cavities with a slightly enlarged VD, mild aortic, mitral, and tricuspid regurgitations, with an estimated systolic pressure in the pulmonary artery of 55 mmHg. Considering the LVEF of 52%, but with a N-terminal pro-brain natriuretic peptide (NT pro-BNP) level of 630 pg/mL, she was classified as having HF with preserved ejection fraction (HFpEF). The treatment was adjusted to include empagliflozin 10 mg/day. Surprisingly, after a while, in parallel with the alleviation of symptoms and signs of HF, we noticed the improvement in VA, which diminished in frequency on the Holter monitoring (302 PVB, without NSVT), allowing the cessation of therapy with amiodarone, see Figure 4.

In May 2024, because the patient’s son was also diagnosed with TSI, a genetic test was performed using a large gene panel sequencing TruSight One Illumina (4813 Genes), but this examination failed to identify a precise diagnosis for the patient’s phenotype.

**Figure 5:**
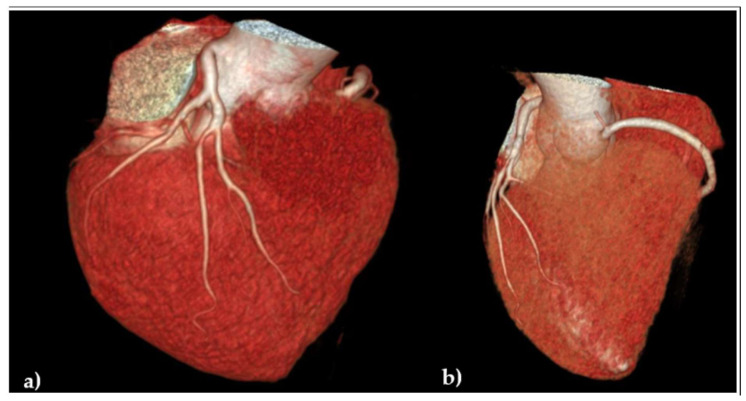
Angio-coronaro-computed tomography: volumetric reconstruction of coronary arteries: (**a**) frontal anterior view evidencing the left coronary artery without significant stenosis; (**b**) transversal cranial view revealing the origin of the right coronary artery.

Considering that the patient continued to claim chest pains, mostly related to physical effort, in spring 2024, an angio-coronary arteries CT was performed, but despite diffuse atheromatosis and small narrowings, it failed to reveal significant coronary artery stenosis, Figure 5.

The last patient’s evaluation, performed in May 2025, revealed isolated PVB and only one doublet on the Holter ECG monitoring, with normal pacemaker function. TTE evidenced no significant alterations compared to previous examinations. Submaximal stress test, stopped at 50 W due to dyspnea, failed to reveal significant ECG changes, except for the negative T waves present before this examination.

This study was conducted in accordance with the Declaration of Helsinki and approved by the Ethics Committee of County Emergency Hospital “Pius Brinzeu” Timisoara (No. 206/7.09.2020). The patient signed an informed consent form.

## 3. Discussion

### 3.1. Association of TSI with Arrhythmias and Conduction Disturbances

Starting from the diagnostic and therapeutic provocations encountered during the management of our case, we investigated the medical literature on well-known databases (Clarivate, PubMed, and Google Scholar). We searched for case reports, published in English as full texts between 2016 to 2025, aiming to identify other case presentations of individuals diagnosed with TSI and SSS, eventually with associated VA, and with similar clinical characteristics and diagnostic challenges as our case, also requiring pacemaker implantation. We decided to select only case reports from the last ten years, when our patient was first diagnosed with arrhythmias, because we assumed a higher likelihood of similar diagnostic and therapeutic approaches. Articles from the grey literature or non-English reports were screened to mitigate selection bias.

During our search of the medical literature, we identified 9 case reports on subjects with TSI and SSS published between 2016 and 2025, as presented in Table 1. It is worth mentioning that, except for the multicenter experience published by Lüker et al., presenting 11 cases of ICD implantation in patients with TSI and VA, and where most of the patients had severe cardiac malformations or cardiomyopathies with heart failure [10], we found no presentation on ventricular tachycardia (VT) occurring in a subject without significant structural cardiac anomaly excepting for dextrocardia.

Intracardiac malformations are diagnosed in 40% to 50% of individuals with TSI, mostly atrial or/and interventricular septal defects, vascular transpositions, or structural anomalies, and even cardiomyopathies. In patients with TSI, LVNC has a 6% higher incidence, compared to subjects without TSI, and similar data are given for AVRC. These malformations are frequently associated with VT [19]. Congenital heart block may also coexist with dextrocardia [20]. There are case reports of early to mid-adulthood presentations of complete heart block in association with dextrocardia [20]. This phenomenon could be due to idiopathic degeneration of the conduction system, congenital conduction system maldevelopment or delayed requirement for cardiac pacing associated with congenital heart block [11]. Other possible hypotheses include abnormal autonomic innervation patterns, or genetic variants affecting laterality and ion channel function [1,3]. Some of the genetic variants responsible for TSI can also increase the risk for arrhythmias through mutations in genes involved in the Nodal signaling pathway (NODAL and LEFTY2) and in ciliary function (DNAH5) [21]. These genetic alterations can influence sarcoplasmic reticular calcium (Ca^2+^) transport and storage properties, which regulate the consequent Ca^2+^–troponin binding, triggering cross-bridge cycling activity and myofilament mechanics, ultimately generating myocardial mechanical ventricular activity [22]. Otherwise, arrhythmias are not so frequently diagnosed in patients with TSI, and little information is available in the medical literature on VA and SSS occurring in patients without structural abnormalities because physicians seldom come face to face with patients with TSI and these comorbidities [9].

Usually, SSS is more often diagnosed in older patients. Thus, an increased incidence of SSS in younger adults, even in their forties, especially in women with TSI, has been described [3,6,13,20], as in the case of our patient, a 49-year-old woman. As presented in Table 1, there is only one 73-year-old man mentioned, who developed SSS after a non-ST elevation acute myocardial infarction [15], the other 8 being women. It can be observed that only 3 of them are over 65 years old, and 2 first developed AF, which was catheter ablated and, subsequently, SSS [14,17]. The youngest patient, a 32-year-old woman with TSI and SSS, was implanted with a DDD device during pregnancy [12]. Considering the younger age of the affected patients, operators may have to perform several pacemaker implantations/battery replacements on a patient with TSI during their whole life.

### 3.2. Challenges of Intracardiac Procedures and Device Implantation in Patients with TSI

Often, SSS in TSI patients manifests with tachy-bradycardia syndrome, combining atrial fibrillation (AF), even in younger subjects [13], and sinus pauses, requiring pacemaker implantation [3,13]. As presented in Table 1, only two cases failed to have AF, as in our patient [9,12]. In patients with TSI and AF with fast ventricular rate, catheter or cryo-ablation are often recommended [22], which, like any other surgical intervention in a patient with TSI, raise difficulties for the operator due to the unusual anatomy and other associated malformations. Zhao et al. performed several successful catheter ablations of AF in patients with TSI by using CT imaging and three-dimensional electro-anatomical mapping, combined with intracardiac echocardiography, and x-ray imaging data [23,24]. Akkaya et al. suggested that cryoballoon-based ablation could be a safe alternative to radiofrequency ablation for atrial arrhythmias originating alongside a pulmonary vein in patients with TSI [8].

In patients with TSI, due to possible malformations at the level of the venous system, the operating team needs to have detailed anatomical information obtained by chest CT or MRI to prepare before surgery [1,24]. Pre- and intraprocedural techniques have to be adapted for this rare type of patient. To ease the implantation procedure, imaging techniques are vital. Some authors suggested inverting the fluoroscopic image to mirror the aspect of a patient without TSI [9]. Several interventional cardiologists recommend venous angiography before intracardiac device implantation, to better evidence the venous route, identification of the optimal venous approach, and adapt the progression of the electrode leads in the right heart cavities, in case of other anomalies [13]. Cine angiograms serve to facilitate lead positioning in the atria and the ventricle and reveal important anatomical information regarding the orientation of the septum, morphology of the venous chambers (whether trabeculated or smooth), and the presence of a venous anomaly. This relates to the interpretation of the anatomical reference points obtained with standard fluoroscopic projections. Other authors used more modern technologies to facilitate the positioning of the leads, like 3D-CT guidance. To detect unknown venous anomalies, this imaging technique can also be used pre-operatively [18].

Usually, operators use the approach from the right side [13], but sometimes, it is useful to ensure bilateral peripheral upper-extremity venous access for situations when the device′s location has to be changed. In contrast to these recommendations, the team that performed the pacemaker implantation on our patient decided to use the left-sided approach, with a good clinical outcome. Fortunately, our patient didn′t have associated venous anomalies, which would have complicated the intervention. It needs to be emphasized that the proximity of the superior vena cava to the left subclavian vein, in the case of mirror image dextrocardia without associated venous anomalies, makes the left venous access a straightforward approach [13]. Some authors suggest using active fixation leads for both the right atria and RV, as it facilitates the best lead positioning. These leads can be fixed to the free wall of the atrium, increasing the chances of obtaining optimal sensing and pacing thresholds [9,16].

In addition, the multicenter study of Lüker et al. debated on the challenges raised by the implantation of a subcutaneous ICD in several patients with dextrocardia and TSI due to the need for new electrode leads implantation, the presence of old, passive, or even fractured leads removal, and sometimes, the necessity of a new device implantation [10].

### 3.3. Genetic Considerations

In our patient, repeated 24 hour ECG Holter monitoring failed to detect AF, but instead, severe VA were evidenced. To our knowledge, all other cases of TSI and VT presented in the medical literature had LVNC [10]. RMN imaging failed to detect ARVC or LVNC in our patient. Although a genetic anomaly was strongly suspected, the TruSight One testing failed to evidence specific abnormalities. Given the familial occurrence of TSI and the limitations of targeted panels such as TruSight One (limited to a broad selection of clinically relevant genes—4813 in total), further genetic evaluation—potentially including whole-exome or whole-genome sequencing—may be warranted to explore variants in genes implicated in laterality defects allowing detection of rare or novel variants in genes implicated in ciliary function, left-right patterning (e.g., DNAI1, ZIC3) and arrhythmia syndromes, acknowledging that a negative result does not totally exclude a genetic etiology as in other pathologies [24,25]. Such approaches may help clarify the genetic basis and refine familial risk assessment. Another particularity of our case is the fact that the EF was normal, although the RV function was slightly impaired. Other authors determined that patients with TSI, who present VT due to LVNC, have reduced EF [10].

### 3.4. Risk Assessment for VA

In patients with TSI and LVNC, epicardial and endocardial mapping and ablation should be performed due to the complexity of the reentrant circuits of VT [26]. In our patient, it was debated on an ICD implantation indication according to the guidelines because there was evidence of frequent episodes of NSVT, and she had a family history of sudden death, all the more so because of the prolonged therapy with amiodarone that resulted in amiodarone-induced hyperthyroidism with its subsequent complications and diagnostic challenges [27]. ICD implantation has been postponed because our patient was oligosymptomatic, never had evidence of syncope or of sustained VT, and the electrophysiological study failed to induce VT, despite comprehensive stimulation protocols, including multi-site pacing, up to three extrastimuli, burst pacing, and adrenergic provocation with high-dose dobutamine. The absence of VT inducibility despite extensive stimulation protocols and adrenergic provocation supports a lower immediate arrhythmic risk, particularly in the context of a negative cardiac MRI and no structural abnormalities. Current recommendations support ICD implantation primarily for patients with sustained VT/VF, hemodynamic instability, or a high-risk structural or genetic substrate. However, we recognize that non-inducibility does not eliminate the potential for future arrhythmic events, especially in patients with suspected inherited arrhythmia syndromes and family history of sudden cardiac death. This underscores the importance of continued clinical monitoring and individualized risk stratification. Another reason for postponing ICD implantation was related to the difficulties raised by the implantation of new electrode leads and devices in patients with TSI due to the inverted and sometimes distorted anatomy and possible venous abnormalities [10,28,29].An interesting aspect of our case was that in 10 years of evolution, in which repeated ECGs and 24 hour Holter monitoring were performed, she was never free of VA despite antiarrhythmic therapy with amiodarone. We only obtained a reduction in the number of NSVT episodes. When amiodarone therapy was withdrawn due to hyperthyroidism, she continued to complain about episodes of palpitations, PVB were present on the ECG, and amiodarone was resumed at a lower dose.

### 3.5. Heart Failure Therapy and Evolution of VA

Surprisingly, after developing HFpEF requiring the addition of guidelines-adjusted therapy with sodium-glucose cotransporter-2 inhibitors (SGLT2i), besides a significant improvement of signs and symptoms characterizing heart failure, we obtained, first, a reduction and, subsequently, the disappearance of VA. However, we have no definitive proof that this improvement can be attributed only to the treatment with SGLT2i, or that it represents a natural evolution of her disease. To our knowledge, this is the first case presentation in the medical literature suggesting that SGLT2i may improve VA burden in patients with TSI. Starting from the hypothesis that SGLT2i may have beneficial direct effects not only on the myocardium by ameliorating its energetic metabolism, reducing oxidative stress, inflammation, fibrosis, and adverse ventricular remodeling, reducing the wall stress, but also on the cardiac ion channels and mediators, favoring potentially antiarrhythmic effects. While there is a consensus in the medical literature concerning the efficiency of SGLT2i on cardiovascular morbidity, hospitalization for heart failure duration and frequency, their antiarrhythmic effects are still a matter of dispute [15,16,22,23]. Several large cohort studies, meta-analyses, and review articles debated the impact of SGLT2i on cardiac arrhythmias, mostly on AF and less on VA, which are believed to be influenced by cardiac electrophysiologic processes by impacting cardiac ion channels and their function [30,31,32,33,34]. However, these hypotheses still remain a matter of debate and require further study.

Study Strengths/Limitations: According to our knowledge, we failed to find another case with TSI and associated complex rhythm and conduction abnormalities in the absence of structural cardiac defects diagnosed by imagistic methods (TTE, CT, and MRI), and without genetic alterations evidenced by genetic testing, by using the (TruSight One). This assessment was performed, but no conclusive results were obtained. This panel contains a broad selection of clinically relevant genes (4813 in total), but further genetic testing (including whole-exome or whole-genome sequencing) may be warranted to explore other gene variants implicated in laterality defects (e.g., DNAI1, ZIC3) and arrhythmia syndromes, acknowledging that a negative result does not exclude a genetic etiology as in other pathologies [24]. Another limitation consists of the failure to reproduce VA during the electrophysiologic study. Thus, we cannot sustain that future arrhythmias will not reappear, or another study could succeed in reproducing them.

## 4. Conclusions/Learning Points

As medical literature data concerning arrhythmias in individuals with TSI are scarce, we considered it important to highlight the challenges raised by an accurate diagnosis and the adapted management of our patient who developed serious arrhythmias in the absence of structural heart disease, as well as the clinical implications. Considering the large spectrum of cardiovascular complications often diagnosed in TSI patients, a comprehensive diagnosis using modern imagistic methods, and genetic testing is an absolute requirement. As peculiar associations of various cardiovascular abnormalities might be diagnosed, a multidisciplinary approach would be necessary for an adapted therapy.

## Figures and Tables

**Table 1:** Selection of case reports of patients with TSI and SSS published between 2016 and 2025.

No	Author/ Year	No of Patients/Gender/age	SSS Form	Particularities	Treatment
1.	Guo et al., 2017 [11]	1/female/50	Bradycardia, sinus pauses, paroxysmal AF	Persistent left superior vena cava	VVI pacemaker
2.	Hayashi et al., 2020 [12]	1/female/32	Bradycardia, sinus pauses	Pregnancy	DDD
3.	Bakalli et al., 2021 [13]	1/female/38	Bradycardia, sinus pauses, paroxysmal AF, junctional escape rhythm	-	DDD
4.	Yokoyama et al., 2021 [14]	1/female/70	Paroxysmal AF, bradycardia, junctional bradycardia	Inferior vena cava occlusion Catheter ablation of AF	DDD
5.	Luo et al., 2022 [9]	1/female/65	Bradycardia, sinus block, PVB, PAB, junctional escape beats	SH	DDD
6.	Bontempi et al., 2022 [15]	1/male/73	Bradycardia, paroxysmal AF with slow and fast ventricular rate, sinus pauses	Non-ST elevation MI, bivascular coronary artery disease with PTCA	Leadless pacemaker
7.	Hakimi et al., 2022 [16]	1/female/43	Bradycardia, junctional bradycardia, Paroxysmal AF	Surgery for severe rheumatic mitral and aortic stenosis	DDD
8.	Khurshid et al., 2023 [17]	1/female/73	Paroxysmal AF, sinus bradycardia	Catheter ablation of AF, interrupted inferior vena cava	DDD
9.	Nomura et al., 2024 [18]	1/woman/75	Bradycardia, sinus pauses, paroxysmal AF	HFpEF	DDD

Legend: AF—atrial fibrillation; DDD—dual chamber pacemaker; HFpEF—heart failure with preserved ejection fraction; MI—myocardial infarction; PAB—premature atrial beats; PTCA—percutaneous coronary angioplasty; PVB—premature ventricular beats; SH—systemic hypertension; SSS—sick sinus syndrome; VVI—ventricular unicameral pacemaker.

## Data Availability

All the data published in this manuscript are available upon request from the first author.

## Abbreviations

The following abbreviations are used in this manuscript:

AF	Atrial fibrillation
AVRC	Arrhythmogenic right ventricular cardiomyopathy
ACE	Angiotensin-converting enzyme
BP	Blood pressure
CT	Computed tomography
DDDR	Dual-chamber rate-responsive
EF	Ejection fraction
HF	Heart failure
HFpEF	HF with preserved ejection fraction
HR	Heart rate
ICD	Intraventricular cardioverter-defibrillator
ECG	Electrocardiogram
LDL	Low-density lipoprotein
LVNC	LV noncompaction
LV	Left ventricular
MRI	Magnetic resonance imaging
NT pro-BNP	N-terminal pro-brain natriuretic peptide
RV	right ventricle
SGLT2i	Sodium-glucose cotransporter-2 inhibitors
SH	systemic hypertension
SSS	Sick sinus syndrome
PVB	Premature ventricular beats
T2DM	Type II diabetes mellitus
TTE	Transthoracic echocardiography
TSI	Total situs inversus
VT	Ventricular tachycardia
